# 

**Published:** 1885-06

**Authors:** 


### Bristol Medico-Chirurgical Society.

### President.

G. F. BURDER, M.D., Aberd.

### President-Elect.

W. H. SPENCER, M.D., M.A. Cantab.

### Honorary Secretary.

R. SHINGLETON SMITH, M.D., B.Sc. Lond.

### Committee.

*Past Presidents.*
R. W. COE.
AUGUSTIN PRICHARD, M.D. Berlin.
W. MICHELL CLARKE.
J. G. SWAYNE, M.D. Lond.
E. LONG FOX, M.D. Oxon.
JAMES G. DAVEY, M.D. St. And.
W. JOHNSTONE FYFFE, M.D. Dub.

F. RICHARDSON CROSS, M.B. Lond.
NELSON C. DOBSON.
L. M. GRIFFITHS.
F. POOLE LANSDOWN.
E. MARKHAM SKERRITT, M.D., B.S., B.A. Lond.
J. GREIG SMITH, M.B., C.M., M.A. Aberd., F.R.S.E.

Medical Men wishing to join the Society are requested to communicate with the Honorary Secretary, Dr. R. SHINGLETON SMITH, 9 Richmond Hill, Clifton. The Annual Subscription is 10/6.

---

*Extracts from Journal By-laws.*

4.—The *Journal* shall be delivered free to Subscribers and also to Members of the Society whose subscriptions are not in arrear.

6.—The Annual Subscription to the *Journal* for those who pay before the 1st of July shall be 5/-, post free, or 6/- if paid after that date.

---

Communications intended for insertion in the *Journal*, Books for Review, &c., should be sent to the Editor, J. GREIG SMITH, M.A., F.R.S.E., 16 Victoria Square, Clifton.

Exchange Journals and communications referring to the delivery of the *Journal* should be sent to the Publication Secretary, L. M. GRIFFITHS, 9 Gordon Road, Clifton, to whom Subscriptions are to be paid *in advance.*

Authors requiring Reprints of their Papers should communicate with the Publisher.

### The following Periodicals are received in Exchange with the Bristol Medico-Chirurgical Journal.

### AFRICA.

CAPE COLONY.

*Monthly.*

*The South African Medical Journal.* East London: T. W. Goodwin.

EGYPT.

*Twice a month.*

*Unione Medica Egiziana.* Alexandria: E. Pergola.

### AMERICA.

ARGENTINE REPUBLIC.

*Monthly.*

*Anales del Circulo Médico Argentino.* Buenos Ayres: M. Biedma.

CANADA.

*Monthly.*

*Canada Medical and Surgical Journal.* Montreal: Gazette Printing Company.

PERU.

*Monthly.*

*La Crónica Médica.* Lima: Carlos Prince.

UNITED STATES.

*Weekly.*

*The Medical and Surgical Reporter.* Philadelphia: 115 South Seventh Street.

*The Medical Record.* New York: William Wood & Co.

*The New York Medical Journal.* New York: D. Appleton & Co.

*The Cincinnati Lancet and Clinic.* Cincinnati: 281 West 7th Street.

*The Journal of the American Medical Association.* Chicago: 65 Randolph Street.

*Fortnightly.*

*Philadelphia Medical Times.* Philadelphia: J. B. Lippincott & Co.

*Monthly.*

*Pacific Medical and Surgical Journal and Western Lancet.* San Francisco: 425 Sutter Street.

*The American Practitioner.* Louisville: John P. Morton and Company.

*The Polyclinic.* Philadelphia: P. Blakiston, Son & Co.

*The Saint Louis Medical and Surgical Journal* Saint Louis: 2622 Washington Avenue.

*The Therapeutic Gazette.* Detroit: George S. Davis.

*The Medical Analectic.* New York: G. P. Putnam's Sons.

*Journal of Cutaneous and Venereal Diseases.* New York: William Wood & Company.

*The American Journal of Ophthalmology.* St. Louis: J. H. Chambers & Co.

*Index Medicus.* Detroit: G. S. Davis.

*The Obstetric Gazette.* Cincinnati: 281 West 7th Street.

*Quarterly.*

*The American Journal of the Medical Sciences.* Philadelphia: Lea Brothers & Co.

*Yearly.*

*Transactions of the American Surgical Association.* Philadelphia: P. Blakiston, Son & Co.

*Occasionally.*

*Transactions of the New York Academy of Medicine.* New York: 12 West 31st Street.

### ASIA.

INDIA.

*Monthly.*

*The Indian Medical Journal.* Allahabad: The Pioneer Press.

JAPAN.

*Monthly.*

*Transactions of the Sei-I-Kwai.* Tokio: Z. P. Maruya & Co.

### AUSTRALASIA.

AUSTRALIA.

*Monthly.*

*The Australasian Medical Gazette.* Sydney: L. Bruck.

*The Australian Medical Journal.* Melbourne: Stillwell & Co.

### EUROPE.

BELGIUM.

*Monthly.*

*Bulletin de l'Académie royale de médecine de Belgique.* Brussels: A. Manceaux.

BRITISH ISLES.

*Weekly.*

*British Medical Journal.* London: 161 A Strand.

*Medical Times and Gazette.* London: J. & A. Churchill.

*The Lancet.* London: 423 Strand.

*Monthly.*

*The Birmingham Medical Review.* Birmingham: Cornish Bros.

*The London Medical Record.* London: Smith, Elder, & Co.

*The Medical Chronicle.* Manchester: John Heywood.

*Edinburgh Medical Journal.* Edinburgh: Oliver and Boyd.

*The Glasgow Medical Journal.* Glasgow: Alex. Macdougall.

*The Dublin Journal of Medical Science.* Dublin: Fannin & Co.

*Quarterly.*

*The Asclepiad.* London: Eade and Caulfield.

*Half-yearly.*

*The Liverpool Medico-Chirurgical Journal.* Liverpool: Medical Institution.

*The Retrospect of Medicine.* London: Simpkin, Marshall, & Co.

*Yearly.*

*The Medical Annual.* London: Henry Kimpton.

*The Year-Book of Treatment.* London: Cassell & Company, Limited.

DENMARK.

*Weekly.*

*Hospitals-Tidende.* Copenhagen: Jacob Lund.

FRANCE.

*Three times a week.*

*Gazette des hôpitaux.* Paris: 4 Rue de l'Odéon.

*L'Union médicale.* Paris: 11 Rue de la Grange-Batelière.

*Weekly.*

*Bulletin de l'Académie de médecine.* Paris: G. Masson.

*Gazette médicale de Paris.* Paris: 8 Place de l'Odéon.

*Le Progrès médical.* Paris: 14 Rue des Carmes.

*Monthly.*

*Revue mensuelle de Laryngologie, d'Otologie et de Rhinologie.* Paris: 8 Place de l'Odéon.

GERMANY.

*Twice a week.*

*Deutsche Medizinal-Zeitung.* Berlin: Eugen Grosser.

*Weekly.*

*Medicinische Bibliographie.* Leipzig: Breitkopf & Härtel.

GREECE.

*Weekly.*

Γαληνὸς. Athens: N. G. Passare.

HOLLAND.

*Weekly.*

*Nederlandsch Tijdschrift voor Geneeskunde.* Amsterdam: F. van Rossen.

ITALY.

*Monthly.*

*Lo Sperimentale.* Florence: 35 Via degli Alfani.

NORWAY.

*Monthly.*

*Norsk Magazin for Lægevidenskaben.* Christiania: Th. Steens.

PORTUGAL.

*Weekly.*

*A Medicina Contemporanea.* Lisbon: José Antonio Rodrigues & Cª

RUSSIA.

*Weekly.*

*Vrach.* St. Petersburg: C. Ricker.

SWITZERLAND.

*Monthly.*

*Illustrirte Monatsschrift der ärztlichen Polytechnik und Centralblatt der orthopädischen Chirurgie.* Berne: K. Schmid.

### Past Presidents

OF THE

### Bristol Medico-Chirurgical Society.

1874—75. FREDERICK BRITTAN, M.D., B.A. Dub.

1875—76. ROBERT WILLIAM COE.

1876—77. SAMUEL MARTYN, M.D. Ed.

1877—78. AUGUSTIN PRICHARD, M.D. Berlin.

1878—79. HENRY EDWARD FRIPP, M.D. Würzburg.

1879—80. WILLIAM MICHELL CLARKE.

1880—81. JOSEPH GRIFFITHS SWAYNE, M.D. Lond.

1881—82. EDWARD LONG FOX, M.D. Oxon.

1882—83. JAMES GEORGE DAVEY, M.D. St. And.

1883—84. WILLIAM JOHNSTONE FYFFE, M.D. Dub

### *BOOKS AND PAMPHLETS RECEIVED.*

From J. & A. Churchill, London:

*Regimen in Gout.* Ebstein. Translated by John Scott. 1885.

*Diagnosis and Surgical Treatment of Abdominal Tumours.* By Sir Spencer Wells, Bart. 1885.

*The Revival of Ovariotomy.* By Sir Spencer Wells. 1885.

*Stricture of the Urethra.* By Sir Henry Thompson. Fourth Edition. 1885.

*Face and Foot Deformities.* By F. Churchill. 1885.

*On Scrofulous Neck and the Surgery of Scrofulous Glands.* By F. C. Allbutt and T. P. Teale. 1885.

*Endemic Goitre or Thyreocele.* By William Robinson. 1885.

From H. K. Lewis, London:

*Common Injuries to Limbs.* By E. Cotterell. 1885.

*Practical Orthopædics.* By H. A. Reeves. 1885.

*Dental Surgery.* By A. W. Barrett. 1885.

From Blakiston & Co., Philadelphia:

*Trans. of Amer. Surg. Association.* Vol. II.

*Surgical Delusions and Follies.* By John B. Roberts. 1884.

From Young J. Pentland, Edinburgh:

*General Botany.* By Behrens. Revised by P. Geddes. 1885.

From F. Van Rossen, Amsterdam:

*Overzicht van de Samenstelling en Pharmacodynamische Waarde der Geneesmiddelen.* By Dr. P. C. Plugge. 1885.

Reprints from Journals, &c.

*Pathology and Treatment of Congenital Clubfoot.* By R. W. Parker and S. G. Shattock.

*Uncontrollable Vomiting of Pregnancy.* By Graily Hewitt.

*Surgical Drainage.* By Geo. J. Robertson.

// Bristol Medico-Chirurgical Journal.

The *Journal* when first published received the following notices:—

"The Editor must be congratulated upon having produced so readable a number for his first issue. The type and illustrations are also to be commended, and we trust that these efforts will meet with the success they deserve."—*Lancet.*

"It contains a large amount of original matter, is well illustrated"—*Medical Record* (New York).

"The book is nicely got up as regards type and paper, and we are glad to notice that a goodly number of practitioners in the neighbourhood have become subscribers. The *Bristol Medico-Chirurgical Journal* has made an excellent start under favourable auspices."—*Medical Times and Gazette.*

"It also contains numerous clinical records, many of which are of a very interesting character."—*Liverpool Medico-Chirurgical Journal.*

"The clinical cases are especially good, and form a strong feature of the number."—*Birmingham Medical Review.*

"The present number is so full of interesting matter that we look forward with pleasure to the appearance of the second."—*Edinburgh Medical Journal.*

"The original papers are . . . . followed by a goodly array of 'clinical records,' book notices, and abstracts. We congratulate the Editor, Mr. J. Greig Smith, on the promising start the new journal seems to have made."—*New York Medical Journal.*

**It is hoped that Subscribers will do all they can to extend the circulation of the Journal, which "is intended to embody and reflect the science and practice of Medicine and Surgery as it exists to-day in the West of England and South Wales."**

